# Establishment of Effective Biomarkers for Depression Diagnosis With Fusion of Multiple Resting-State Connectivity Measures

**DOI:** 10.3389/fnins.2021.729958

**Published:** 2021-09-09

**Authors:** Yanling Li, Xin Dai, Huawang Wu, Lijie Wang

**Affiliations:** ^1^School of Electrical Engineering and Electronic Information, Xihua University, Chengdu, China; ^2^Key Laboratory of Fluid and Power Machinery, Ministry of Education, Xihua University, Chengdu, China; ^3^Key Laboratory of Fluid Machinery and Engineering, Sichuan Province, Xihua University, Chengdu, China; ^4^School of Automation, Chongqing University, Chongqing, China; ^5^The Affiliated Brain Hospital of Guangzhou Medical University (Guangzhou Huiai Hospital), Guangzhou, China; ^6^School of Computer Science and Engineering, University of Electronic Science and Technology of China, Chengdu, China

**Keywords:** fusion, resting-state functional connectivity, effective connectivity, dynamic functional connectivity, classification

## Abstract

Major depressive disorder (MDD) is a severe mental disorder and is lacking in biomarkers for clinical diagnosis. Previous studies have demonstrated that functional abnormalities of the unifying triple networks are the underlying basis of the neuropathology of depression. However, whether the functional properties of the triple network are effective biomarkers for the diagnosis of depression remains unclear. In our study, we used independent component analysis to define the triple networks, and resting-state functional connectivities (RSFCs), effective connectivities (EC) measured with dynamic causal modeling (DCM), and dynamic functional connectivity (dFC) measured with the sliding window method were applied to map the functional interactions between subcomponents of triple networks. Two-sample *t*-tests with *p* < 0.05 with Bonferroni correction were used to identify the significant differences between healthy controls (HCs) and MDD. Compared with HCs, the MDD showed significantly increased intrinsic FC between the left central executive network (CEN) and salience network (SAL), increased EC from the right CEN to left CEN, decreased EC from the right CEN to the default mode network (DMN), and decreased dFC between the right CEN and SAL, DMN. Moreover, by fusion of the changed RSFC, EC, and dFC as features, support vector classification could effectively distinguish the MDD from HCs. Our results demonstrated that fusion of the multiple functional connectivities measures of the triple networks is an effective way to reveal functional disruptions for MDD, which may facilitate establishing the clinical diagnosis biomarkers for depression.

## Introduction

Major depressive disorder (MDD) is a severe mental illness with emotional and cognitive abnormalities, and anhedonia, reduced energy, poor attention, and concentration are core symptoms of MDD (Diener et al., 2012; Belzung et al., 2015). Recently, the triple network model, consisting of the central executive network (CEN), default mode network (DMN), and salience network (SAL), was proposed, and dysfunctions of the three networks may underlay the cognitive and affective abnormalities in psychiatric and neurological disorders (Menon, 2011). Although the functional abnormalities of the three networks have been reported in different studies (Kaiser et al., 2015; Mulders et al., 2015; Brakowski et al., 2017; Wang et al., 2017c), it remains unclear whether/how the intrinsic functional changes and the casual influences between the sub-components of the three networks contribute to the neuropathology of depression.

Resting-state functional connectivity (RSFC), which can be used to investigate the temporal coherence of spontaneous neural activity, offers a task-free approach to detect the intrinsic functional brain networks (Yeo et al., 2011; Wang et al., 2015, 2017d; Glasser et al., 2016; Wang et al., 2019). Independent component analysis (ICA) is a model-free method to obtain a set of components that are maximally independent of each other (Calhoun et al., 2009). ICA has been widely used to define large-scale brain networks, such as DMN, CEN, SAL, and visual and motor networks, in a large number of previous studies (van den Heuvel and Hulshoff Pol, 2010; Mulders et al., 2015; Luo et al., 2021). To explore the causal effects between brain regions, effective connectivity (EC) is a valuable method to identify the information flow during functional interaction (Wang et al., 2017b; Wang et al., 2020). Dynamic causal modeling (DCM) is able to estimate the causal influences of one neuronal subpopulation over another to characterize the causal organization of the brain (Friston et al., 2013). Moreover, more and more studies applied dynamic functional connectivity (dFC) using a sliding window method to reveal the time dynamic of functional couplings between brain areas (Allen et al., 2014). Thus, using intrinsic, effective, and dynamic connectivities to explore the abnormal interactions between the sub-components of the tripe network without any assumption may provide us with new information specific to the neuropathology of MDD. In addition, fusion of the multiple functional connectivity measures may facilitate establishing more effective diagnosis biomarkers than using single connectivity measures.

In this study, we first applied ICA to define the triple network and to extract the time courses of each sub-network using the resting-state fMRI data in 27 MDD patients and 28 healthy controls (HCs). Next, the RSFC, EC, and dFC between each pair of sub-components were analyzed and compared to HC and MDD to identify the group differences. Finally, the changed connectivity measures were taken as features to set up the classification models for MDD to identify the diagnosis biomarkers.

## Materials and Methods

### Subjects

In total, 27 drug-free MDD patients and 28 HC subjects were recruited, and written informed consent was provided and obtained from each subject. MDD patients were diagnosed with the Structured Clinical Interview for DSM Disorders (SCID) using DSM-IV criteria, and the severity of depressive symptoms was measured by Hamilton Depression Rating Scale (HAMD). The inclusion criteria for MDD patients were as follows: not taking any antidepressant medication during the recurrent episode; not having any other comorbid mental disorders; and no contraindications showing up on MRI scans. The HC subjects were also included, and the exclusion criteria were as follows: known personal or family history of psychiatric disorders; current or lifetime diagnosis of Axis I illness; lifetime history of substance abuse or dependence, head trauma, seizures, serious medical or surgical illness; or contraindications showing up on MRI scans. The current study was approved by the Ethics Committee of The Affiliated Brain Hospital of Guangzhou Medical University.

### Resting-State fMRI Data Acquisition

Resting-state fMRI data acquisition was performed using a 3.0-Tesla Philips MR imaging system with an eight-channel SENSE head coil and echo-planar imaging (GRE-EPI) sequence. Before the scanning, all subjects were asked to relax, keep their eyes closed, and not fall asleep. The detailed scanning parameters were as follows: repetition time (TR) = 2000 ms, echo time (TE) = 30 ms, flip angle (FA) = 90^*o*^, field of view (FOV) = 220 × 220 mm^2^, matrix = 64 × 64, slice thickness = 4 mm, inter-slice gap = 0.6 mm, and volume of 240.

### Resting-State fMRI Preprocessing

The resting-state fMRI data were preprocessed using SPM8 software^1^ with various steps, including discarding the first 10 volumes, head motion correction, spatial normalization to the standard EPI template, and smoothing with a 6 mm Gaussian kernel. For resting-state functional and EC analyses, the time courses of each subcomponent of the triple network obtained by ICA were further detrended, despiked, and filtered with a bandpass of 0.01–0.1 Hz.

### Group ICA

The spatial group ICA was used to identify the different resting-state components in all MDD patients and HCs using the GIFT toolbox^2^ (Calhoun et al., 2001; Erhardt et al., 2011; Calhoun and Adali, 2012). The principal component analysis was first used to reduce the dimensions of the functional data. Next, the number of independent components was automatically estimated using the Infomax algorithm to define the most stable and reliable components by running them 100 times with the ICASSO algorithm (Bell and Sejnowski, 1995), and 28 components were finally found. Then, subject-specific time series and spatial ICs were back reconstructed and converted into *z*-maps (Calhoun et al., 2001; Erhardt et al., 2011). Finally, the sub-components of the triple network were identified by visually checking all the independent components for subsequent analyses. The detailed procedures for ICA analysis can be found in our previous study (Luo et al., 2021).

### Functional Network Connectivity (FNC) Analysis

The RSFCs between sub-components of the triple network were calculated. Next, a Fisher *r*-to-*z* transformation was applied to convert the correlation coefficient to *z* values to improve normality. Finally, two-sample *t*-tests were performed to identify the significant alterations in FCs between MDD and HCs. The significance level was set at *p* < 0.05 with Bonferroni corrections.

### DCM Analyses

To calculate the EC, the time series for each sub-component of the triple network was first obtained as state above. Then, the spectral DCM (dcm), which is developed specifically for resting-state fMRI DCM analyses, was used to investigate the causal interaction between the sub-components of the triple network in both MDD and HCs. The spDCM is an extension of the conventional DCM except, adding a stochastic term and removing the modulatory component. This means that spDCM estimates the time-invariant covariance between time series instead of estimating time-varying hidden states. Thus, spDCM only needs to estimate the covariance of the random fluctuations, a scale-free (power law) form for the state noise. The detailed procedures for spDCM can be found in a previous study (Razi et al., 2015). After obtaining the ECs for each subject, two-sample *t*-tests were used to compare the causal effects between MDD patients and controls. The significant level was set at *p* < 0.05 with Bonferroni correction.

### dFC Analyses

The dFC was calculated using a sliding window method. Since the length of the sliding window is the absence of a standard criterion, the length of the sliding window was set at 1/*f*_*min*_ (*f*_*min*_ is the minimum frequency of time series), which has been proven to be able to well characterize the time dynamics (Leonardi and Van De Ville, 2015; Du et al., 2017; Li et al., 2019). Thus, a window length of 50 TR (100 s) with a step size of 5 TR (10 s) as the optimal parameter was applied to keep the balance between capturing reliable dynamics and obtain steady correlations between regions. In each window, the FC values were computed between any pair of subcomponents of triple networks, and the variance of the FC values across all the windows was used to measure the dynamic. Finally, the dFC values were normalized to z-scores for statistical analyses.

### Correlation Analyses

Pearson correlation analyses were conducted between the changed FNC, EC, dFC, and HAMD scores and disease duration. The significance was set at a threshold of *p* < 0.05. No correction was performed to show the trend of the associations because of the small samples in our study.

### SVM Classification

To validate whether multiple connectivity measures could serve as effective biomarkers for depression, fusions of changed RSFC, EC, and dFC were taken as features, and a linear support vector classification (SVC) was employed to train the mode for classifying (Chang and Lin, 2011). A leave-one-out cross-validation (LOOCV) test was used to assess the generalization ability because of the limited number of samples in the present study. The classification result was assessed using the classification accuracy, sensitivity, specificity, and area under the curve (AUC) values.

## Results

### Demographics and Clinical Characteristics

The demographics and clinical characteristics of the HCs and MDD patients are shown in Table 1. There are no significant differences in gender (*p* = 0.66), age (*p* = 0.63), and education level (*p* = 0.94) between MDD and HCs.

**TABLE 1:** Demographics and clinical characteristics ofthe used subjects.

**Subjects**	**MDD**	**HC**	***P*-value**
Number of subjects	27	28	
Gender (male: female)	10/17	12/16	0.66
Age (mean ± SD)	29.67 ± 7.26	30.57 ± 6.68	0.63
Years of education (mean ± SD)	13.83 ± 3.70	13.89 ± 2.2	0.94
HDRS scores (mean ± SD)	33.56 ± 7.21		
Age of onset (years)	26.48 ± 7.82		
Duration of illness(months)	38.92 ± 54.96		

*A Pearson chi-squared test was used for gender comparison. Two-sample *t*-tests were used for age and education comparisons. HDRS, Hamilton Depression Rating Scale score; MDD, major depression disorder (MDD); HC, healthy control (HC).*

### ICA Results

**FIGURE 1:**
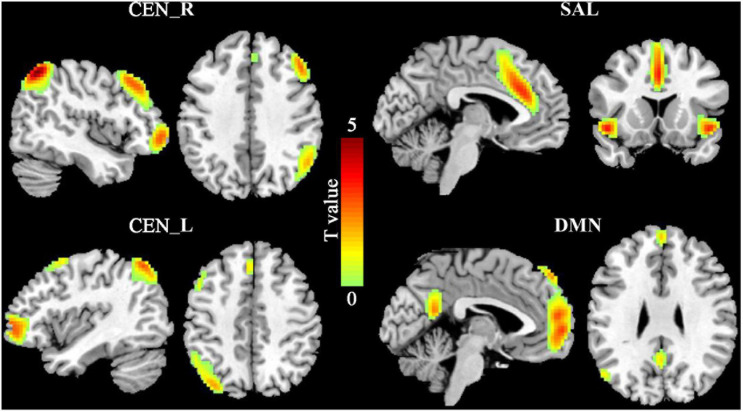
The triple networks. The group independent component analysis (ICA) was used to define the triple networks. Four subcomponents of the triple networks were identified including default mode network (DMN), salience network (SAL), and left and right central executive network (CEN_L, CEN_R).

Four sub-components of the triple network including left and right CEN (CEN_L, CEN_R), DMN, and SAL were identified in this study (Figure 1). The spatial patterns of the four subcomponents of the triple network were consistent with the previous findings (Damoiseaux et al., 2006; Arbabshirani et al., 2013; Mueller et al., 2014).

### Resting-State FNC Results

**FIGURE 2:**
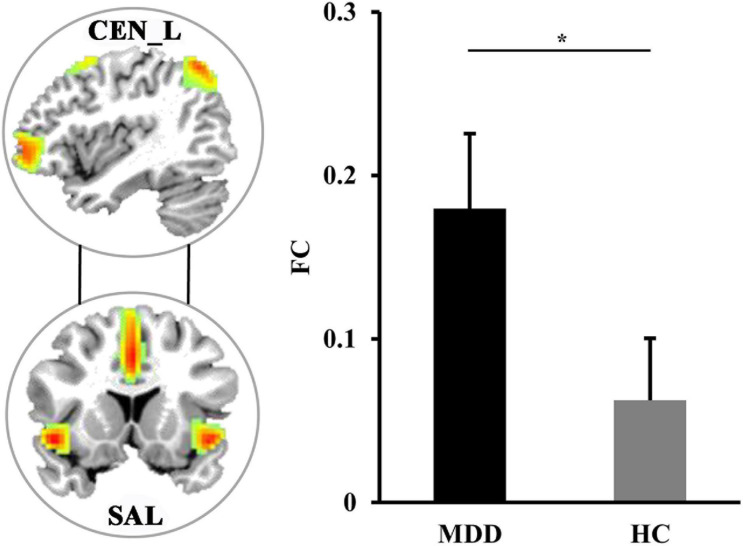
The differences in functional network connectivities. The significantly increased functional network connectivity between the left central executive network (CEN_L) and salience network (SAL) was found in MDD patients. *Represents significant difference.

Pearson correlation coefficients between each pair of the four sub-components were calculated to study the changes of the large-scale FC. Compared with HCs, the MDD patients had significantly increased FC between left CEN (CEN_L) and SAL (*p* = 0.0082) (Figure 2).

### DCM Results

**FIGURE 3:**
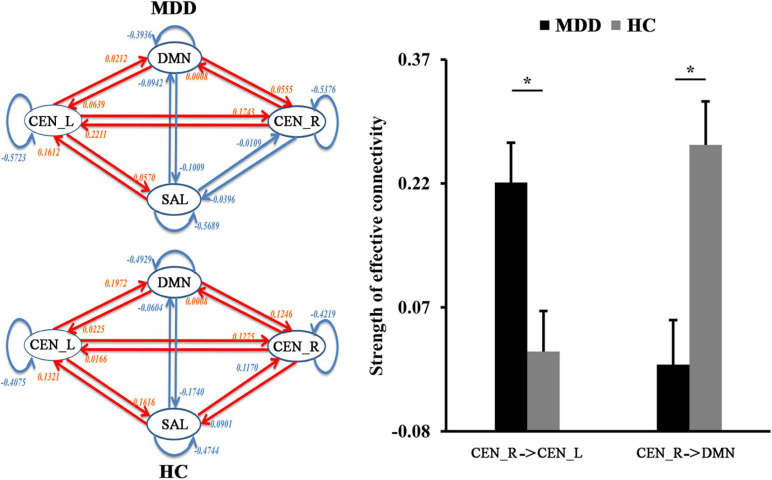
The differences in functional network effective connectivities. Dynamic causal modeling was used to determine the differences in effective connectivities between the triple networks. The significantly increased effective connectivity from right to the left central executive network (CEN) was found in MDD patients while significantly decreased effective connectivity from right CEN to default mode network (DMN) was found in MDD patients. *Represents significant difference.

The spDCM was performed to identify the changes of casual interactions between sub-components of the triple network in MDD. Compared with HCs, the significantly increased magnitude of causal interactions from right CEN (CEN_R) to CEN_L (*p* = 0.0045) and significantly decreased magnitude of causal interactions from the right CEN_R to DMN (*p* = 0.00087) were found in MDD patients (Figure 3).

### dFC Results

**FIGURE 4:**
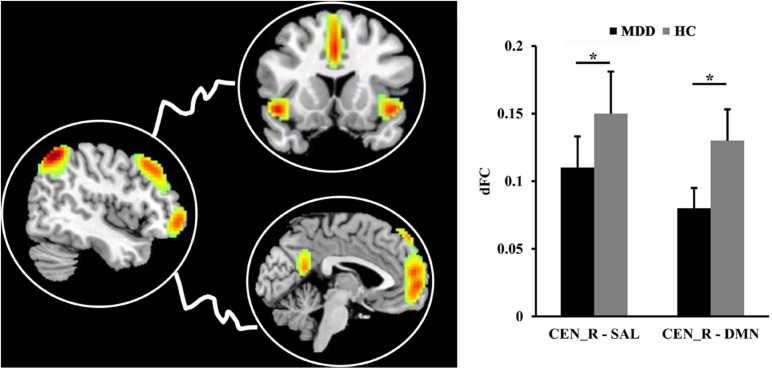
The differences in dynamic functional connectivity (dFC). The significantly decreased dFC between right central executive network (CEN) and salience network (SAL), default mode network (DMN) was found in MDD patients compared to healthy controls. *Represents significant difference.

The significantly decreased dFC between right CEN (CEN_R) and SAL (*p* = 0.012), DMN (*p* = 0.011) were found in MDD patients as compared to HCs (Figure 4).

### Clinical Correlations

**FIGURE 5:**
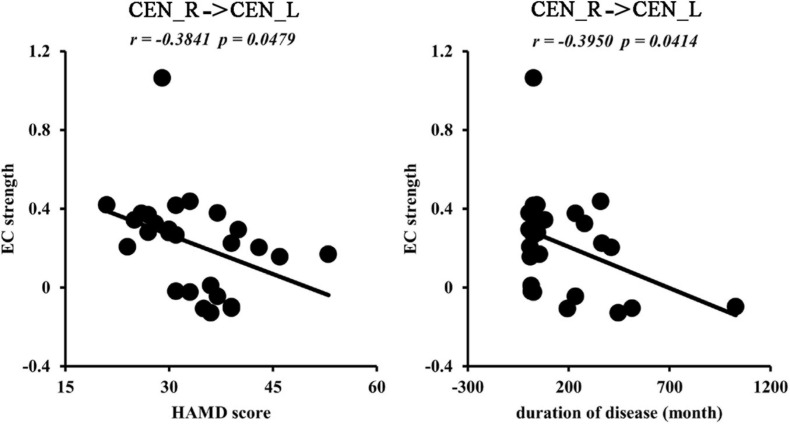
Correlation analyses. The significantly negative correlations between effective functional connectivities from right to left central network networks (CEN) and Hamilton Depression Rating Scale (HAMD) scores, disease duration were found in MDD patients.

We found negative correlations between the ECs from CEN_R to CEN_L and HAMD scores (*r* = −0.3841, *p* = 0.0479) and disease duration (*r* = −0.3950, *p* = 0.0414) (Figure 5).

### Classification Results

**FIGURE 6:**
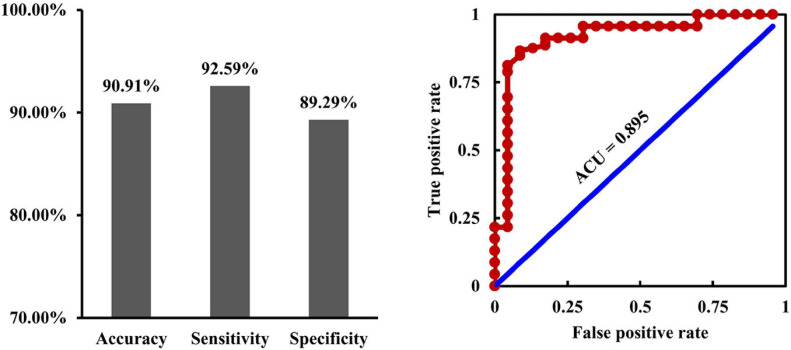
Fusion of connectivity measures for classification. By fusion of the changed resting-state, functional connectivities, causal effective connectivities, and dynamic functional connectivities as features, support vector classification could effectively differentiate depression from healthy controls with an accuracy of 90.91%, a sensitivity of 92.59%, a specificity of 89.29%, and an ACU of 0.895.

With the fusion of changed RSFC, EC, and dFC as features, SVC could distinguish MDD from HCs with an accuracy of 90.91%, a sensitivity of 92.59%, a specificity of 89.29%, and an ACU of 0.895 (Figure 6).

## Discussion

In this study, we aimed to explore the intrinsic, effective, and dynamic connectivity alterations between subcomponents of the triple networks to reveal the potential neuropathology of MDD. Compared to HCs, MDD patients showed increased intrinsic functional connectivity between CEN_L and SAL, increased EC from CEN_R to CEN_L, decreased EC from CEN_R to DMN, and decreased dFC between CEN_R and SAL, DMN. Interestingly, the increased ECs from CEN_R to CEN_L were negatively correlated with HAMD scores and disease duration in MDD patients. Furthermore, by fusion of the multiple connectivity measures, we demonstrated that changed RSFC, EC, and dFC could effectively distinguish MDD from HCs. Our findings provide evidence for how functional disorganization of the triple network in MDD patients could facilitate the development of clinical diagnosis biomarkers for depression.

We found abnormal functional interactions among CEN, SAL, and DMN in MDD patients with increased functional couplings between left CEN and SAL, decreased EC from CEN to DMN, and decreased dFC between CEN and SAL, DMN in MDD patients. Our findings were consistent with that reported in MDD patients in previous studies (Greicius et al., 2007; Zhu et al., 2012; Wang et al., 2017a, 2018). SAL plays an important role in switching information between CEN and DMN (Menon and Uddin, 2010; Menon, 2011). CEN is mainly involved in external executive and cognitive control, while DMN is mainly involved in internal attention and self-reference (Corbetta and Shulman, 2002; Hamilton et al., 2015; Wang et al., 2015, 2016; Wu et al., 2016; Wang et al., 2019). The increased functional connections between CEN and SAL may be a compensatory mechanism for the functional impairments in switching between external and internal attention in MDD patients (Barch and Sheffield, 2014). On the contrary, the decreased EC from CEN to DMN and dFC between CEN and SAL, DMN indicated disrupted switching between the internal self-reference and the demand cognitive action (Seeley et al., 2007; Scheibner et al., 2017). All the evidence suggested that functional dysfunctions of information switching among CEN, SAL, and DMN may be the neuroanatomical basis of rumination of MDD. Moreover, we found that the changed RSFC, ECs, and dFC as features could effectively distinguish the MDD patients from HCs. This finding indicated that the abnormal functional couplings of the triple network may be the underlying neuropathological mechanism of depression.

Interestingly, our study revealed increased EC from the right CEN to left CEN in MDD patients, and the effective connections were closely associated with depression symptoms and disease duration. This finding indicated that the functional balance of bilateral CEN is fundamental to maintaining the normal functions of the brain in MDD patients (Grimm et al., 2008; Triggs et al., 2010; Chen et al., 2013). Moreover, we found that the effective connections were negatively correlated with HAMD scores. This finding suggests that enhanced interaction from the right to left CEN is a compensatory mechanism and not a neuropathological change.

There are some limitations to our study. First, the sample size in our study is relative small, and a larger number of patients are needed to validate the findings in further studies. Second, although all the patients are medication-free in the current episode, some patients took antidepressant medications before. Thus, the first-episodic drug-naïve MDD patients are warranted to better identify the neural basis for MDD.

## Conclusion

This study revealed large-scale functional network dysfunctions in MDD, including increased functional connectivity between left CEN and SAL, increased EC from right CEN to left CEN, reduced EC from right CEN to DMN, and decreased dFC between right CEN and SAL, DMN. Moreover, by fusion of the changed connectivity measures as features, our study revealed that it is able to distinguish MDD from HCs. These findings provide new evidence for the neuropathology of triple networks in MDD. Our study may facilitate developing clinical diagnosis biomarkers and the future treatment for MDD.

## Data Availability Statement

The raw data supporting the conclusions of this article will be made available by the authors, without undue reservation.

## Ethics Statement

The studies involving human participants were reviewed and approved by the Ethics Committee of The Affiliated Brain Hospital of Guangzhou Medical University. The patients/participants provided their written informed consent to participate in this study.

## Author Contributions

All authors listed have made a substantial, direct and intellectual contribution to the work, and approved it for publication.

## Conflict of Interest

The authors declare that the research was conducted in the absence of any commercial or financial relationships that could be construed as a potential conflict of interest.

## Publisher’s Note

All claims expressed in this article are solely those of the authors and do not necessarily represent those of their affiliated organizations, or those of the publisher, the editors and the reviewers. Any product that may be evaluated in this article, or claim that may be made by its manufacturer, is not guaranteed or endorsed by the publisher.

## Footnotes

1
https://www.fil.ion.ucl.ac.uk/spm/software/spm8/

2
http://mialab.mrn.org/software/gift
